# The PP4R1 sub-unit of protein phosphatase PP4 is essential for inhibition of NF-κB by merkel polyomavirus small tumour antigen

**DOI:** 10.18632/oncotarget.15836

**Published:** 2017-03-02

**Authors:** Hussein Abdul-Sada, Marietta Müller, Rajni Mehta, Rachel Toth, J. Simon C. Arthur, Adrian Whitehouse, Andrew Macdonald

**Affiliations:** ^1^ School of Molecular and Cellular Biology, Faculty of Biological Sciences, Astbury Centre for Structural Molecular Biology, University of Leeds, Leeds, UK; ^2^ Division of Immunology and Cell Signalling and Division of Signal Transduction Therapy College of Life Sciences, University of Dundee, Dundee, UK

**Keywords:** virus, skin cancer, NF-κB, immune evasion, phosphatase

## Abstract

Merkel cell carcinoma (MCC) is a highly aggressive skin cancer with a high metastatic potential. The majority of MCC cases are caused by the Merkel cell polyomavirus (MCPyV), through expression of the virus-encoded tumour antigens. Whilst mechanisms attributing tumour antigen expression to transformation are being uncovered, little is known of the mechanisms by which MCPyV persists in the host. We previously identified the MCPyV small T antigen (tAg) as a novel inhibitor of nuclear factor kappa B (NF-kB) signalling and a modulator of the host anti-viral response. Here we demonstrate that regulation of NF-kB activation involves a previously undocumented interaction between tAg and regulatory sub-unit 1 of protein phosphatase 4 (PP4R1). Formation of a complex with PP4R1 and PP4c is required to bridge MCPyV tAg to the NEMO adaptor protein, allowing deactivation of the NF-kB pathway. Mutations in MCPyV tAg that fail to interact with components of this complex, or siRNA depletion of PP4R1, prevents tAg-mediated inhibition of NF-kB and pro-inflammatory cytokine production. Comparison of tAg binding partners from other human polyomavirus demonstrates that interactions with NEMO and PP4R1 are unique to MCPyV. Collectively, these data identify PP4R1 as a novel target for virus subversion of the host anti-viral response.

## INTRODUCTION

Merkel Cell Carcinoma (MCC) is a rare and highly malignant neuroendocrine carcinoma of the skin with a high level of metastasis and poor five-year survival rate [1, 2]. In 2008, the DNA of a novel member of the *Polyomaviridae*, Merkel cell polyomavirus (MCPyV), was detected in MCC samples [3]. Epidemiological studies demonstrate that viral DNA is clonally integrated in approximately 80% of MCC investigated [3]. In keeping with other polyomaviruses, MCPyV expresses early proteins including the large (TAg) and small (tAg) tumour antigens, and a number of splice variants thereof [4]. These function as regulatory proteins and are essential for virus replication and pathogenesis. MCPyV isolated from MCC contains a truncated form of LT that retains the amino terminus but lacks the carboxyl-terminus helicase domain and is defective for virus replication [3]. MCPyV T-antigens are essential for MCC cell survival and proliferation, and depletion of either protein using siRNA leads to cell cycle arrest and apoptosis [4]. In agreement with these observations, mice genetically engineered to express MCPyV T antigens in the stratified epithelium display signs of neoplastic progression including unscheduled DNA synthesis, increased cellular proliferation and evidence of DNA damage. These data support the theory that MCPyV T antigens are oncogenic [5]. Unlike Simian Virus 40 (SV40), MCPyV tAg expression is sufficient to transform rodent fibroblasts to contact- and anchorage-independent growth, and allows serum independent proliferation of human cells [6]. A number of host binding partners are required for MCPyV functions [1, 7]. For example, MCPyV tAg binding to the ubiquitin ligase complex SCF^Fbw7^ prevents the ubiquitin-dependent degradation of TAg and a number of host cell oncoproteins [8]. Mutational studies identified a stretch of 5 amino acids (91-95) within tAg, termed the LT-stabilisation domain (LSD), to be essential for this function [8]. Expression of MCPyV tAg also results in the hyperphosphorylation of the translational regulator 4E-BP1, resulting in the deregulation of cap-dependent translation [6]. The cellular microtubule network is destabilized in cells expressing tAg, and this is associated with reduced phosphorylation of the microtubule regulatory protein Stathmin [9]. Targeting this pathway enhances MCC migration and invasion, which may explain the highly metastatic nature of MCPyV-associated MCC [7]. Expression of tAg also perturbs the host anti-viral immune response by inhibiting activation of the nuclear factor kappa B (NF-κB) transcription factor [10]. Down-regulation of this pathway likely contributes to the chronic nature of MCPyV infection. Similar to other polyomaviruses, tAg binds to members of the protein phosphatase family of enzymes, including PP2A Aα, PP2A Aβ and PP4c [10, 11]. Whilst the role of the PP2A Aα interaction is not clear, mutational studies implicate PP2A Aβ and PP4c in the control of NF-κB activation [10].

NF-κB is a family of transcription factors that play essential roles in the host response to infection [12]. The family consists of five structurally homologous members that form dimers sequestered in the cytoplasm of unstimulated cells, via an interaction with inhibitor of κB (IκB) proteins [13]. NF-κB is activated in response to a wide range of factors including pro-inflammatory cytokines (e.g. tumour necrosis factor alpha (TNFα)) or engagement of pattern recognition receptors (PRRs) by their ligands (e.g. dsRNA or hypomethylated DNA). Receptor activation initiates downstream signalling events that culminate in activation of the IκB kinase (IKK) complex [14]. This complex consists of two catalytic sub-units, IKKα and IKKβ, and a third, non-catalytic, adaptor protein termed IKKγ or NF-κB essential modulator (NEMO). NEMO is a 419 amino acid protein containing two coiled-coil domains (CC1 and CC2), a ubiquitin-binding in ABIN and NEMO (UBAN) domain, a leucine zipper (LZ) and zinc finger domain [15]. IKKα/β are each bound constitutively to a NEMO dimer through residues 50-86 in NEMO. Recruitment of NEMO to hybrid linear/Lysine-(K)63-linked polyubiquitin chains brings the IKK complex into proximity of TAK1, which phosphorylates and activates the IKK complex [16]. Activated IKK phosphorylates IκBα, resulting in its rapid ubiquitin-dependent degradation, allowing the released NF-κB dimer to translocate to the nucleus and activate the transcription of genes associated with inflammation and the anti-viral response.

Our group identified a functional interaction between tAg and NEMO in cells and showed that this interaction was necessary for the inhibition of NF-κB dependent transcription in MCC cell lines [10]. Polyomaviruses mediate many of their effects through their manipulation of host protein phosphatases [11, 17]. Whilst MCPyV tAg targets PP2A Aα through direct protein-protein interactions, mutations in tAg that impair this interaction (e.g. R7A) do not prevent inhibition of NF-κB activation [10]. Instead, using a SILAC based quantitative proteomic approach; we identified a novel interaction with the catalytic sub-unit of the PP4 phosphatase. This interaction was essential for the reduction in IKK activity observed in MCPyV tAg expressing cells. Whilst tAg and NEMO may interact in a protein complex, the molecular basis by which tAg associates with NEMO is unclear. In this study, we show that NEMO is not a direct binding partner of tAg. Rather, we identify PP4 and its regulatory sub-unit, PP4R1, as necessary for generating a tAg-NEMO complex. Using specific tAg mutants able to discriminate between distinct host phosphatase sub-units, and an siRNA depletion approach, we validated the importance of PP4R1 for inhibition of NF-κB by MCPyV. Finally, screening with tAg from a number of polyomaviruses showed that binding to PP4R1 and NEMO was unique to MCPyV. Together, these data indicate that MCPyV utilizes unique interactions with cellular phosphatases to inhibit a critical antiviral pathway.

## RESULTS

### Mutations in the UBAN domain of NEMO prevent the interaction with MCPyV tAg in cells

**Figure 1:**
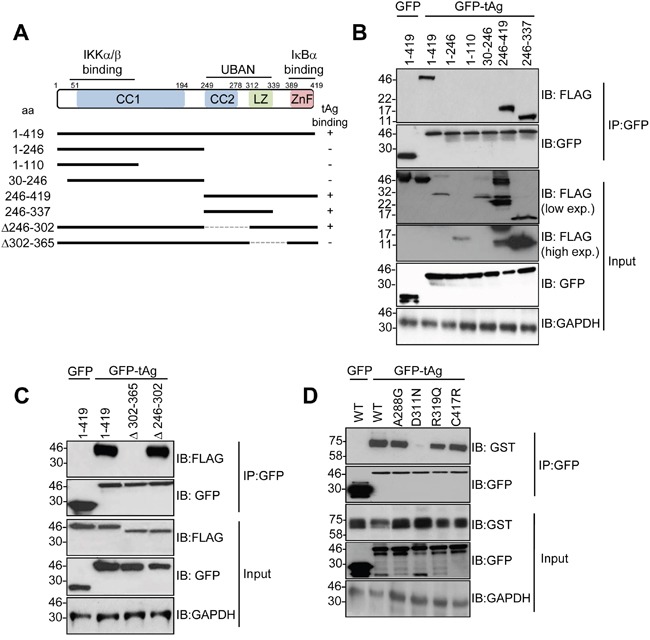
The NEMO UBAN domain is necessary for tAg binding in cells **(A)** Schematic representation of the FLAG-tagged NEMO truncations and internal deletions. **(B)** MCC13 cells were transfected with plasmids encoding GFP fusion proteins and a panel of FLAG-tagged NEMO truncations. GFP-TRAP co-immunoprecipitations were performed on the transfected lysates, and both input lysates and precipitations were probed with antibodies against GFP and FLAG. Lysates probed with an antibody detecting GAPDH served as a loading control. **(C)** GFP-TRAP co-immunoprecipitations were performed with a GFP control or GFP MCPyV tAg and two FLAG-tagged internal deletions of NEMO. Precipitations and lysates were analyzed as above. **(D)** MCC13 cells were transfected with plasmids encoding GFP fusion proteins and GST-tagged NEMO point mutants. GFP-TRAP co-immunoprecipitations were performed on the transfected lysates, and both lysate and precipitation were probed with antibodies against GFP and GST. GAPDH served as a loading control. Western blots shown are representative from at least three independent experimental repeats.

We previously demonstrated that tAg interacts with NEMO in cells [10]. To investigate the mechanisms mediating this interaction, we firstly generated NEMO deletion mutants containing the region responsible for IKKα/β binding, the UBAN domain and the zinc finger (Figure 1A). All truncation mutants expressed to varying levels in MCC13 Merkel cell carcinoma cells (Figure 1B). Co-immunoprecipitation experiments showed that tAg only interacted with full-length NEMO (1-419) and truncations containing the UBAN domain (e.g. NEMO 246-419). NEMO truncations consisting of the amino terminal domain, necessary for binding the IKK complex, failed to interact with tAg (e.g. NEMO 1-246). To confirm these results, two internal deletion mutants of NEMO were generated in which regions of the coiled-coil domain (NEMO Δ246-302) or leucine zipper (NEMO Δ302-365) were removed. Whilst these internal deletions expressed to wild-type levels, only the coiled-coil domain deletion bound to tAg at similar levels to wild type NEMO, whereas the leucine zipper deletion mutant abrogated the interaction with tAg (Figure 1C). Several studies have identified mutations within this region of NEMO in patients suffering from immunodeficiencies [18, 19]. A small number of characterized mutants were generated, known to disrupt specific functions of NEMO [18, 19]. Two mutations were generated in the UBAN domain, D311N and R319Q. Mutation of either residue has been shown to abrogate the binding of NEMO to ubiquitin and to prevent activation of NF-κB in response to a number of stimuli [20]. In addition, two mutants were generated outwith the UBAN domain to act as controls in the experiment. An A288G mutation in NEMO affects the second coiled-coil domain and destabilizes NEMO oligomers, resulting in impaired activation of NF-κB [21]. Finally, a C417R substitution modifies the structure of the carboxyl-terminal zinc finger and impairs activation of NF-κB in response to CD40 ligation [22]. After confirming that all four mutants expressed to comparable levels as wild type NEMO, we next compared their ability to interact with tAg using co-immunoprecipitation assays. Interestingly, we found that NEMO containing the R319R substitution within the UBAN domain could bind tAg, but the D311N substitution failed to interact with tAg (Figure 1D). As expected, based on results obtained using the NEMO internal deletions, substitution of A288G or C417R had negligible impact on binding to tAg.

### NEMO does not interact directly with MCPyV tAg *in vitro*

**Figure 2:**
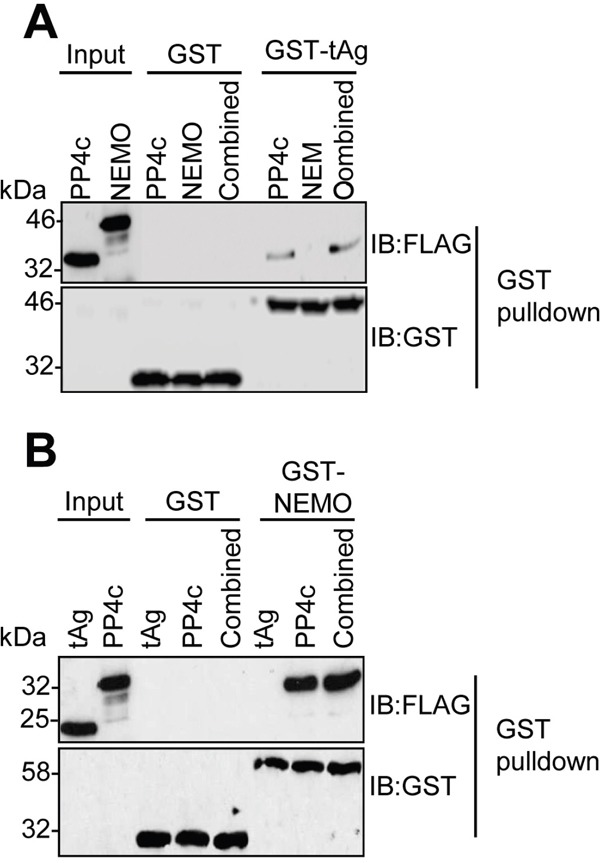
MCPyV tAg interacts with PP4c but not NEMO *in vitro* **(A)** Equal amounts of bacterially expressed GST and GST-tAg were bound to glutathione-agarose beads and incubated with ITT produced FLAG-tagged PP4c and NEMO alone or in combination. Following washes, bound proteins were separated by SDS PAGE and probed with antibodies against GST and FLAG. A sample of the ITT input was analyzed to confirm appropriate expression of the FLAG-tagged proteins. **(B)** Equal amounts of bacterially expressed GST and GST-NEMO were bound to glutathione-agarose beads and incubated with ITT produced FLAG-tagged PP4c and tAg alone or in combination. Following washes, bound proteins were separated by SDS PAGE and probed with antibodies against GST and FLAG. A sample of the ITT input was analyzed to confirm appropriate expression of the FLAG-tagged proteins. Western blots are representative of at least three independent repeats.

We previously demonstrated that tAg interacts with NEMO and the catalytic sub-unit of the PP4 phosphatase (PP4c) in cells [9, 10]. To understand the mechanism of these interactions, we wished to determine whether tAg interacted directly with NEMO or PP4c. For this, NEMO and PP4c were generated by *in vitro* coupled transcription/translation (ITT), and used in GST pull-down experiments with bacterial expressed GST-tAg [23]. Analysis showed that, in contrast to PP4c, GST-tAg did not bind directly to NEMO (Figure 2A). It was possible that tAg might recruit NEMO in a PP4c-dependent manner; therefore, we next determined whether co-incubation with NEMO and PP4c would recapitulate the tAg-NEMO interaction we had observed in cells. GST pull-downs were performed in mixed reactions containing ITT produced NEMO and PP4c. Even with the presence of PP4c, GST-tAg was not able to bind to NEMO *in vitro*. Reciprocal experiments using a bacterial expressed GST-NEMO as bait for interaction with ITT expressed PP4c or tAg were in agreement, showing only an interaction between NEMO and PP4c (Figure 2B). These data determine that tAg binds to PP4c *in vitro* but that the interaction between tAg and NEMO must be mediated through an additional interaction beyond PP4c.

### PP4R1 is a novel tAg binding partner

**Figure 3:**
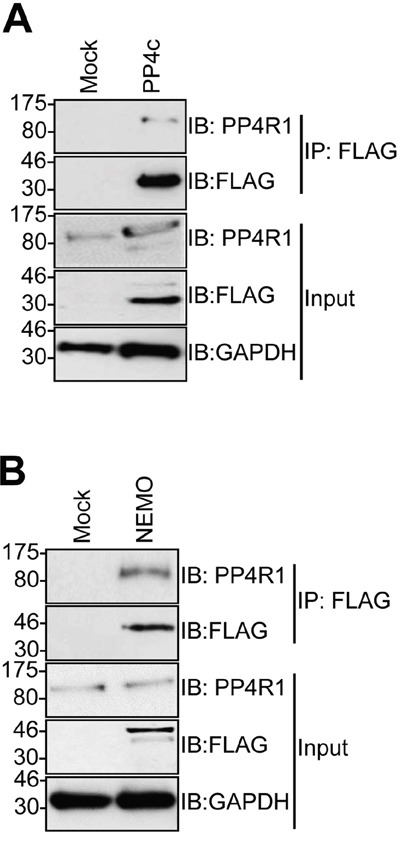
PP4R1 interacts with PP4c and NEMO in MCC13 cells MCC13 cells were transfected with empty plasmid or FLAG-tagged **(A)** PP4c or **(B)** NEMO. Immunoprecipitations were performed using FLAG-agarose beads and analyzed by western blot with antibodies against FLAG or endogenous PP4R1. Total cell lysates served as a positive control for expression. Western blots shown are representative of at least three independent experiments.

**Figure 4:**
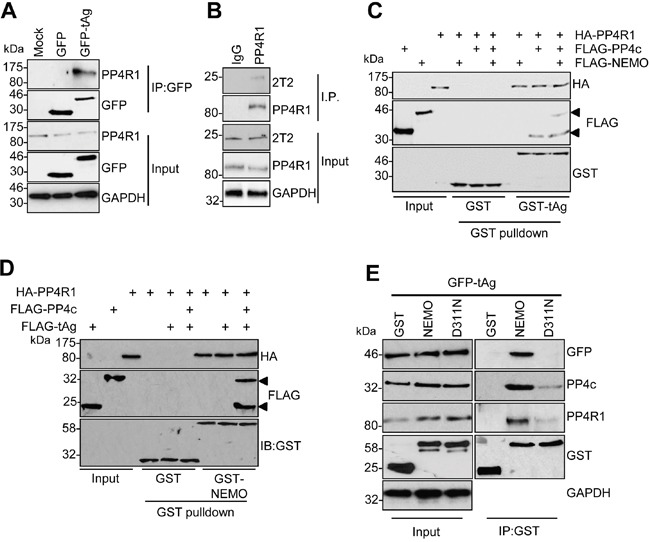
PP4R1 is a novel tAg binding partner required for NEMO binding **(A)** GFP-TRAP co-immunoprecipitations were performed on lysates from MCC13 cells transfected with plasmids expressing GFP or GFP-tAg and analyzed by western blot with antibodies against GFP and endogenous PP4R1. Total cell lysates served as a positive control for protein expression and GAPDH as a loading control. **(B)** MKL1 cell lysates were precipitated with an anti-PP4R1 antibody or a pre-immune IgG control and analyzed by western blot. Samples were probed with antibodies against PP4R1, tAg (2T2) and GAPDH served as a loading control. **(C)** Equal amounts of bacterially expressed GST and GST-tAg were bound to glutathione-agarose beads and incubated with ITT produced HA-PP4R1 and FLAG-PP4c/NEMO alone or in combination. Following washes, bound proteins were separated by SDS PAGE and probed with antibodies against GST, HA and FLAG. A sample of the ITT input was analyzed to confirm appropriate expression of the epitope-tagged proteins. **(D)** Equal amounts of bacterially expressed GST and GST-NEMO were bound to glutathione-agarose beads and incubated with ITT produced HA-PP4R1 and FLAG-PP4c/tAg alone or in combination. Following washes, bound proteins were separated by SDS PAGE and probed with antibodies against GST, HA and FLAG. A sample of the ITT input was analyzed to confirm appropriate expression of the epitope-tagged proteins. **(E)** MCC13 cells were transfected with plasmids expressing GST, GST-NEMO or GST-NEMO D311N in combination with GFP-tAg. Cell lysates were incubated with glutathione-agarose beads and precipitates probed with antibodies against GST, GFP, PP4c and PP4R1. Total cell lysates served as an expression control and GADPH as a loading control. Western blots shown are representative of at least three independent experimental repeats.

The failure of PP4c to recapitulate an interaction between tAg and NEMO *in vitro* suggested that a further host protein partner was necessary to allow tAg to complex with NEMO. PP4c has been shown to associate with the non-catalytic protein phosphatase 4 regulatory sub-unit 1 (PP4R1) in the cytoplasm of cells [24]. Given the crucial role of these scaffolding sub-units in phosphatase function, we set out to determine whether PP4R1 might be required for tAg-mediated inhibition of NF-κB. Initially, we evaluated whether endogenous PP4R1 was able to interact with PP4c and NEMO in MCC13 cells. FLAG-tagged versions of PP4c and NEMO were precipitated from MCC13 cells and western blot analysis showed that endogenous PP4R1 interacted with both proteins (Figure 3A and 3B). Next, we explored whether PP4R1 interacts with tAg. Firstly, MCC13 cells were transfected with GFP or GFP-tAg and precipitations performed using GFP-TRAP beads. Results show that endogenous PP4R1 bound to GFP-tAg but not GFP alone (Figure 4A). To ensure that the interaction observed was not a result of tAg over-expression, lysates were generated from MKL1 cells, a MCPyV positive MCC tumour cell line. Lysates were next precipitated with an anti-PP4R1 antibody or a pre-immune IgG control. Tumour expressed tAg was successfully precipitated with endogenous PP4R1 but not with the pre-immune IgG control (Figure 4B). These data demonstrate that tAg interacts with PP4R1 in a MCPyV positive MCC cell line. Together, these data provide the first evidence of a viral protein associating with PP4R1.

### PP4R1 is required for the interaction between tAg and NEMO

Having established that tAg, PP4c and NEMO associate with PP4R1 in cells, we sought to determine whether PP4R1 functions as an adaptor in the tAg-NEMO complex. To this end, we repeated *in vitro* binding assays using bacterial expressed GST-tAg (Figure 4C) or GST-NEMO (Figure 4D) and ITT produced binding partners. As before, PP4c but not NEMO bound directly to recombinant GST-tAg. PP4R1 was also able to bind directly to GST-tAg and GST-NEMO *in vitro* but, similar to PP4c, could not recapitulate the tAg-NEMO interaction. Only when PP4R1-PP4c and NEMO were combined was GST-tAg able to bind to NEMO in the *in vitro* assay (Figure 4C). If the interactions observed between tAg and NEMO observed in cells are facilitated by PP4c-PP4R1, then mutations in the NEMO UBAN domain that impair the interaction with tAg (Figure 1D) should not interact with PP4c-PP4R1. To investigate this possibility, co-immunoprecipitations were performed from cells expressing GST-NEMO or GST-NEMO D311N and endogenous PP4c-PP4R1. In agreement with our previous result, introduction of the single point mutation into the UBAN domain of NEMO abrogated the interaction with GFP-tAg (Figure 4E). This mutation also significantly decreased binding to endogenous PP4c and PP4R1, further suggesting that these phosphatase sub-units are recruited to the IKK complex via interactions with NEMO.

### Mapping of tAg residues necessary for interacting with NEMO and phosphatase sub-units

**Figure 5:**
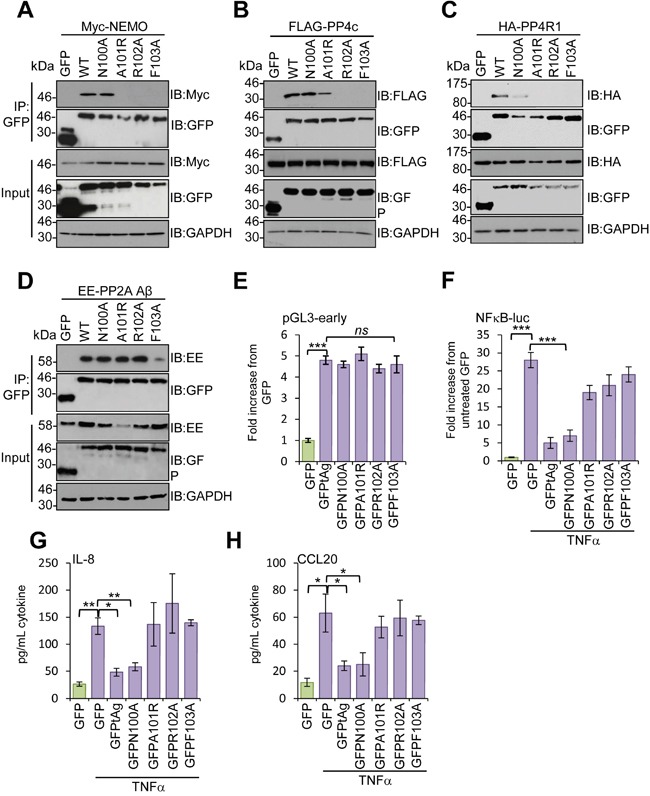
Mapping the residues in MCPyV tAg necessary for inhibition of NF-κB activation GFP-TRAP co-immunoprecipitations were performed using MCC13 cells co-transfected with plasmids expressing GFP, GFP-tAg or a panel of tAg point mutants in the presence of **(A)** a Myc-NEMO expression plasmid, **(B)** FLAG-PP4c expression plasmid, **(C)** HA-PP4R1 expression plasmid or **(D)** EE-tagged PP2A Aβ expression plasmid. Transfected cell lysates were incubated with GFP-TRAP affinity beads and bound protein was western blotted with antibodies detecting GFP and the appropriate epitope tag. Total cell lysates served as a positive control for protein expression and GAPDH as a loading control. **(E)** MCC13 cells were transfected with plasmids encoding the GFP-fusion proteins described and a luciferase reporter plasmid encoding the MCPyV early promoter [10]. Cell lysates were harvested after 24 hours and used in luciferase assays. **(F)** MCC13 cells were transfected with plasmids expressing GFP-fusion proteins and a reporter plasmid driving firefly luciferase under the control of NF-κB elements from the Concanavalin A promoter. Cells were treated with 10 ng/mL TNFα for 8 hours and samples analyzed for luciferase activity. To normalize for differences in transfection efficiency cells were co-transfected with a renilla luciferase reporter plasmid, and values were normalized relative to renilla expression. **(G)** IL-8 and **(H)** CCL20 protein levels in TNFα stimulated cells. Cells were stimulated as in **(F)**, and media collected at 24 hours post stimulation for analysis by ELISA for each cytokine. Data are presented as picograms of cytokine. Error bars are +/−SD. Significance was analyzed by student's t-test and is indicated by an asterix *p<0.05, **p<0.01, ***p<0.001. Data shown are representative of at least three independent experimental repeats.

Studies using deletion analysis indicated that tAg bound to NEMO and the phosphatases PP2A Aβ and PP4c through a 16 amino stretch (residues 95 -111) [10]. Consistent with this, an internal deletion mutant of tAg lacking these residues was incapable of blocking NF-κB mediated transcription [10]. To determine which residues within this region were required for interaction with NEMO and the phosphatase sub-units, we undertook a mutagenesis screen in which residues were mutated to alanine, or existing alanines changed to arginine, in sets of 4. We then investigated the effect of these mutations on the ability of tAg to bind to NEMO, PP4c, PP4R1 and PP2A Aβ. Most mutations did not affect the binding; however, mutation of N100-F103 prevented binding to all proteins under study (data not shown). To investigate further, point mutations were generated in this region and tested for interactions with the panel of binding partners. Mutation of N100 had no significant impact on binding to NEMO or PP4c, although some reduction was observed for PP4R1, which likely reflects the reduced levels of the GFP-N100A protein. Mutation of A101, R102 or F103 disrupted binding of tAg to both NEMO and PP4R1, whilst binding to PP4c was diminished upon mutation of A101 and abrogated in the R102-F103 mutants (Figure 5A-5C). In contrast, the interaction with PP2A Aβ was only lost in the F103A mutant (Figure 5D). Importantly, all of the point mutants retained the ability to stimulate expression from the MCPyV early promoter, up-regulating the promoter reporter plasmid by approximately 5-fold, similar to wild-type tAg, compared with a GFP control (Figure 5E). These results indicate that the point mutations retain other tAg functions. We next determined the effect of the point mutants on the ability of tAg to down-regulate NF-κB-driven transcription using a luciferase reporter system. As seen previously, tAg expression strongly inhibited NF-κB-driven luciferase expression in response to treatment with TNFα (Figure 5F). A similar level of inhibition was observed for the N100A mutant, which retained binding to NEMO and the phosphatases. In contrast, tAg with mutations in A101-R102 showed a reduced ability to inhibit NF-κB transactivation, which was fully restored to control levels in the F103 mutant (Figure 5F). To verify the results using a more physiologically relevant indicator, we analyzed the media from mock and treated samples for levels of the pro-inflammatory cytokine IL-8 and the chemokine CCL20 (Figure 5G and 5H). As NF-κB-dependent gene products, their expression was increased upon stimulation with TNFα and their relative levels reflected the data obtained using the luciferase reporter plasmid system.

### PP4R1 is crucial for tAg-mediated inhibition of NF-κB activation

**Figure 6:**
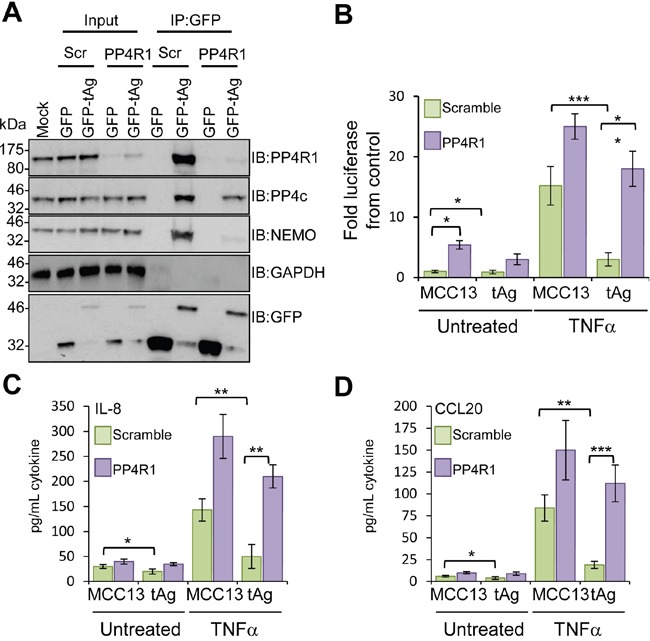
PP4R1 is necessary for MCPyV tAg-mediated inhibition of NF-κB **(A)** Plasmids expressing GFP or GFP-tAg were transfected into MCC13 cells pre-transfected with scrambled or PP4R1-specific siRNA and GFP-TRAP co-immunoprecipitations performed. Precipitated protein complexes were separated by SDS PAGE and western blot performed with antibodies against GFP and endogenous PP4R1, PP4c and NEMO. Total lysates served as positive controls for protein expression, and confirmed PP4R1 depletion, whilst GAPDH acted as a loading control. **(B)** MCC13 cells containing scrambled control or PP4R1-specific siRNA were transfected with plasmids expressing GFP or GFP-tAg and a reporter plasmid driving firefly luciferase under the control of NF-κB elements from the Concanavalin A promoter. Cells were treated with 10 ng/mL TNFα for 8 hours and samples analyzed for luciferase activity. To normalize for differences in transfection efficiency cells were co-transfected with a renilla luciferase reporter plasmid, and values were normalized relative to renilla expression. **(C)** IL-8 and **(D)** CCL20 protein levels in TNFα stimulated cells. Cells were stimulated as in **(B)**, and media collected at 24 hours post stimulation for analysis by ELISA for each cytokine. Data are presented as picograms of cytokine. Error bars are +/−SD. Significance was analyzed by student's t-test and is indicated by an asterix *p<0.05, **p<0.01, ***p<0.001. Data shown are representative of at least three independent experimental repeats.

To substantiate a role for PP4R1 in perturbation of NF-κB activation by tAg, NEMO-tAg complex formation was measured in addition to NF-κB-dependent production of pro-inflammatory cytokines in cells depleted of PP4R1. MCC13 cells were transfected with scrambled or PP4R1-specific siRNAs, and after 24 hours further transfected with either GFP or GFP-tAg and incubated for a further 24 hours prior to cell lysis and analysis of tAg interacting partners. Immunoblotting with an antibody detecting endogenous PP4R1 confirmed efficient knockdown (Figure 6A). PP4R1 depletion had no overall impact on levels of PP4c or NEMO expression. GFP immunoprecipitations were performed from siRNA transfected cell lysates, and the precipitates probed with antibodies against GFP, PP4R1, PP4c and NEMO. As expected, GFP-tAg successfully precipitated endogenous NEMO, PP4R1 and PP4c from scramble siRNA treated cells (Figure 6A). In contrast, absence of PP4R1 abrogated the interaction between GFP-tAg and NEMO, whilst leading to a reduction in the level of PP4c bound (Figure 6A), which confirms that PP4R1 is essential to direct tAg to NEMO in cells.

Our data indicates that PP4R1 directs tAg-PP4c to NEMO in order to inhibit the IKK complex. To further address this point, NF-κB-dependent luciferase reporter assays were performed in tAg expressing cells depleted of PP4R1. As expected, NF-κB activation was increased in PP4R1 depleted cells compared to the scramble control and NF-κB activation was further augmented when cells were treated with TNFα (Figure 6B). Importantly, the impairment of NF-κB activation by tAg was lost both in control and TNFα treated cells. Silencing of PP4R1 significantly increased IL-8 and CCL20 protein production in MCC13 cells upon treatment with TNFα (Figure 6C-6D). Although basal levels of cytokine were increased marginally in the absence of PP4R1, the increase was not to the extent observed upon stimulation. In agreement with the luciferase reporter assay studies, tAg-mediated suppression of pro-inflammatory cytokine protein levels was significantly reduced in the absence of PP4R1. Collectively, these results show that PP4R1 is essential for the suppression of NF-κB signalling by tAg in MCC13 cells.

### Interactions with PP4R1 and NEMO are restricted to MCPyV tAg

**Figure 7:**
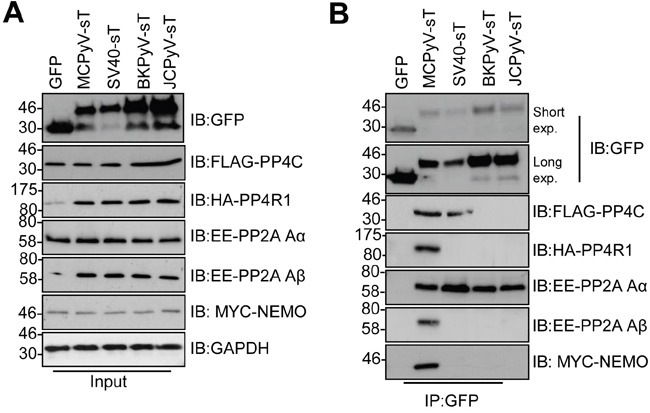
Binding to NEMO and PP4R1 is not conserved amongst PyV tAg **(A)** Total lysates from MCC13 cells transfected with plasmids expressing GFP and GFP-tAg from MCPyV, SV40, BKPyV and JCPyV in the presence of FLAG-PP4c, HA-PP4R1, EE-PP2A Aα, EE-PP2A Aβ or Myc-NEMO expression plasmids. GAPDH served as a loading control. **(B)** GFP-TRAP co-immunoprecipitations performed from cell lysates and western blots probed with antibodies against GFP and the appropriate epitope tag. Western blots shown are representative of five independent experimental repeats.

Given its essential nature, we wished to determine whether the tAg of other human polyomaviruses interact with NEMO and protein phosphatases. We investigated three additional polyomaviruses, namely BK and JC, which are human pathogens and SV40. MCC13 cells were co-transfected with GFP-tAg and epitope tagged versions of NEMO and a panel of phosphatase sub-units (Figure 7). Despite the variations in tAg expression, all tAg tested precipitated PP2A Aα, whereas only MCPyV interacted with PP2A Aβ, as previously published [10, 25]. Our results also confirmed the recently published interaction between SV40 tAg and PP4c [11]. Interestingly, the tAg of SV40, JC and BK did not bind to PP4R1 or NEMO. Only MCPyV was able to precipitate PP4R1 and NEMO from cells. These data further indicate that an interaction between tAg and PP4c alone is not sufficient to mediate an interaction with NEMO in cells.

## DISCUSSION

Detection of viral infection by components of the innate immune system activates signalling cascades that lead to the production of interferons and inflammatory cytokines, in order to eliminate the infection [12]. Amongst these, NF-κB is crucial for activating inflammatory immune responses [13, 26]. Binding of the adaptor protein NEMO to upstream proteins is a critical signalling node that links stimulated receptors to activation of the IKK complex. Subsequent phosphorylation of inhibitory IκB proteins by the IKK complex is a key step in the activation of NF-κB family members and critically represents a focal point of classical NF-κB signalling. Given its importance, many viruses have evolved to target components of the IKK complex to suppress NF-κB activation [27–30]. We previously described that MCPyV tAg was able to prevent IKK phosphorylation by a mechanism requiring interactions with the IKK adaptor protein NEMO and the catalytic sub-unit of PP4 [10]. Dephosphorylation is an essential regulatory element in the NF-κB pathway; however, the identity and control of phosphatases that regulate IKK are not well defined. Deletion analysis showed that the amino terminus of NEMO, required for interactions with IKKα/β, was dispensable for tAg binding. Rather, we demonstrated that the NEMO UBAN domain was necessary for the observed interaction. The UBAN is a coiled-coil rich domain shown to preferentially bind to linear ubiquitin chains, and also form lower affinity complexes with longer K-63 linked polyubiquitin chains [15, 20, 31, 32]. UBAN is essential for recruiting NEMO to upstream signalling complexes and specific point mutations in this domain prevent TNFα and IL-1-mediated NF-κB activation [33]. Co-immunoprecipitations with a panel of NEMO proteins containing point mutants associated with immunodeficiency disorders reinforced the importance of the UBAN domain for the interaction with tAg. Using a point mutant that affects a conserved aspartic acid (D311N) within this domain, identified in X-linked anyhydrotic ectodermal dysplasia with immunodeficiency, prevented the interaction with tAg in cells. The mutation of this residue prevents NEMO from binding to linear and K-63 linked ubiquitin chains and abrogates recruitment to upstream signalling complexes [34]. Interestingly, the R319Q mutation, which also resides within the UBAN domain, did not impact on the interaction with tAg. This mutant was shown to still partially interact with ubiquitin chains and to have a less pronounced phenotype than the D311N mutant [35]. These data indicate that the interaction between tAg and NEMO requires an intact UBAN domain, which may be necessary to facilitate recognition of specific protein complexes or ubiquitylated interaction partners. We further explored tAg-NEMO binding requirements using a combination of recombinant GST-tAg/NEMO and *in vitro* transcribed/translated binding partners. Surprisingly, in contrast with co-immunoprecipitation data, no interaction was observed between tAg and NEMO, suggesting that NEMO is not a direct binding partner of tAg. This might explain the failure to detect NEMO in the initial tAg SILAC proteomic screen [10]. In contrast, PP4c was readily precipitated by both NEMO and tAg. These findings suggest that an essential linker protein may be required to bridge NEMO to tAg. PP4c was an obvious candidate for this role, given its co-localization with NEMO and tAg in discrete cytoplasmic puncta [10], however, introduction of PP4c into the binding assays failed to recapitulate a tAg-NEMO interaction *in vitro*. Given that MCPyV tAg interacts with a range of phosphatase sub-units [10, 11], it was possible that an alternative phosphatase might function as the bridge to NEMO. A number of lines of evidence suggested that this was unlikely. Firstly, whilst PP2A has been shown to dephosphorylate proteins within the NF-κB pathway, a tAg mutant (R7A) unable to interact with PP2A Aα was still capable of inhibiting NF-κB activation [10]. Secondly, a growing body of evidence identifies PP4 as a key negative regulator of NF-κB [36–38]. A range of regulatory sub-units controls the catalytic activity of PP4. These constrain the broad acting catalytic sub-unit activity by restricting its subcellular locations and directing it towards specific substrates [39]. Recently, the regulatory sub-unit PP4R1 has been shown to negatively regulate NF-κB signalling in T cells by directing PP4c to dephosphorylate the IKK complex [37]. We confirmed that endogenous PP4R1 interacted with NEMO and PP4c in MCC13 cells, suggesting that similar inhibitory protein complexes might form in these cells. Importantly we also confirmed that PP4R1 was a novel binding partner of tAg in MCPyV positive cells. To our knowledge, this is the first evidence for a virus protein targeting this phosphatase sub-unit. PP4R1 also bound to both NEMO and tAg *in vitro* but was not sufficient to recapitulate the NEMO-tAg interaction. Only the combined presence of PP4R1-PP4c recapitulated the NEMO-tAg interaction *in vitro*. Together, these binding studies suggest that whilst tAg is not able to bind with NEMO *in vitro*, it interacts with a PP4R1-PP4c phosphatase complex, which mediates the interaction with NEMO to dephosphorylate the IKK complex. In support of this idea, the NEMO UBAN mutant that does not bind with tAg in cells was also less able to interact with PP4R1-PP4c in co-immunoprecipitation assays. Whether the NEMO UBAN interacts with an ubiquitylated form of PP4R1-PP4c or if it has an additional function as a protein interaction domain is not currently known. Importantly, PP4R1 recruits tAg to NEMO, and absence of PP4R1 prevented co-precipitation of NEMO with tAg in cells. Although PP4c was still detected in tAg precipitates in PP4R1 depleted cells, levels were reduced compared to a scrambled control. This suggests that tAg is able to interact with PP4c in the absence of PP4R1; however, presence of the targeting sub-unit enhances the interaction in cells. Depletion of PP4R1 increased levels of basal and stimulus-dependent NF-κB activity, and increased the production of the pro-inflammatory cytokines IL-8 and CCL20, whilst also preventing tAg-mediated inhibition of the pathway. These data confirm reports that PP4R1 is necessary for the shutdown of NF-κB activity, and importantly show that its loss prevents tAg subversion of this pathway.

Whilst there is evidence for binding to a range of phosphatase sub-units by MCPyV tAg, the molecular mechanisms and physiological outcome of many of these interactions are unclear. In this regard, the use of point mutations to abrogate the interaction with specific phosphatases has been illuminating. For example, the R7A mutation prevents interaction with PP2A Aα, and this mutant has allowed the exclusion of a role for PP2A Aα in the regulation of 4EBP1 hyperphosphorylation and in tAg mediated transformation [11]. In contrast, generation of mutants able to discriminate between PP2A Aβ and PP4c has been lacking. Alanine scanning allowed us to develop the first tAg mutants able to selectively prevent binding to PP4c whilst retaining an interaction with PP2A Aβ. Using these mutants we were able to verify that loss of PP4c binding correlated with the loss of NF-κB transcriptional inhibition, as measured by luciferase reporter assay and ELISA detection of pro-inflammatory cytokine production. In the future, these mutants will provide a valuable resource to investigate which phosphatase sub-units are necessary for the range of functions ascribed to tAg, including modulation of the host factor Stathmin [9] or 4EBP1 hyperphosphorylation [6]. Interestingly, the PP4R1-NEMO interaction was not conserved with tAg of other human polyomaviruses, indicating that down-regulation of NF-κB might be a feature only found in this virus. In support of this notion, the NARF sequence residing in residues 100-103 is not found in other family members, including the closely related Chimpanzee and Gorilla PyV. The unique wide range of phosphatase binding partners observed for MCPyV offers an exciting model whereby distinct pools of tAg exist in the cell, each bound by a sub-set of host factors and able to perform specific tasks during the infectious life cycle and in disease progression. Future work should focus on delineating these processes in greater detail.

Collectively, our data provide further evidence that tAg is a suppressor of NF-κB activation, which likely impairs the local innate immune response to MCPyV infection. We have identified an unexplored role for the PP4R1 phosphatase sub-unit in the inhibition of this pathway.

## MATERIALS AND METHODS

### Plasmids, siRNAs and antibodies

Expression plasmids for enhanced green fluorescent protein (GFP)-MCPyV tAg, glutathione S-transferase (GST)-NEMO and Myc-NEMO have been described previously [10]. EE-PP2A A, FLAG-PP4c and HA-PP4R1 were kindly provided by Stefan Stack, Marilyn Goudreault and Arnold Rüdiger, respectively. pGFP-MCPyV tAg N100A, A101R, R102A and F103A, were produced using the QuikChange site directed mutagenesis kit (Stratagene), as directed by the manufacturer's instructions. tAg were PCR amplified from genomic clones of SV40, BKPyV and JCPyV kindly provided by Eric Blair and Michael Imperiale. DNA inserts were cloned between the Eco RI and Bam HI sites of pGFP-C1. Truncation and internal deletion mutants of NEMO were generated by PCR and cloned between Bam HI and Not I restriction sites in pCMV5-FLAG. Point mutations in the UBAN domain of NEMO (A288G, D311N, R319Q and C417R) were generated with QuikChange using pEBG2T-NEMO as template. Bacterial expression of a GST-NEMO fusion protein was achieved by cloning NEMO into pGEX6P1 as a Bam HI and Not I fragment. The MCPyV early promoter region was PCR amplified from MCC genomic DNA using primers incorporating KpnI and SmaI restriction sites, allowing the promoter to be inserted 5′ of the luciferase gene in pGL3-Basic, to generate pGL3-Early [10]. Primer sequences are available on request. All inserts were sequence verified (GATC). PP4R1-specific small interfering RNAs (siRNAs) were purchased from Qiagen. Antibodies against PP4c, PP4R1, GAPDH (Santa Cruz), GFP, NEMO, Glu-Glu (EE) (Abcam), FLAG, GST and Myc (Sigma-Aldrich) were purchased from their respective suppliers. The 2T2 hybridoma, which detects MCPyV tAg was kindly provided by Chris Buck.

### Cells

MCC13 (MCC positive, MCPyV negative) and MKL1 (MCC positive, MCPyV positive) cells were purchased from the ECACC and confirmed to be negative for Mycoplasma infection. Cells were cultured in RPMI 1640 medium supplemented with 10% FBS and 1% penicillin-streptomycin in 5% CO_2_ at 37°C. MCC13 cells were transfected using Lipofectamine 2000 (Invitrogen) as described [10].

### Western blotting

Cells were lysed in Leeds Lysis Buffer (LLB) [40] supplemented with protease inhibitor cocktail (Roche). Proteins were separated by SDS PAGE before transfer onto nitrocellulose membrane (HyBond, Amersham BioSciences) using a TransBlot Turbo (BioRad). Membranes were probed with appropriate primary antibody and horseradish conjugated secondary antibodies. Proteins were detected using ECL (Amersham BioSciences). Western blot analysis was carried out as previously described [10].

### Immunoprecipitations

Assays were performed as previously described [41]. For epitope tag-based co-immunoprecipitations, MCC13 cells were transfected with the appropriate plasmids. Cell lysates were harvested and then incubated with GFP-TRAP M beads (Chromotek) or anti-FLAG M1 affinity gel (Sigma-Aldrich) for 2-4 hours at 4°C or 1 hour at room temperature with vigorous shaking. Beads were washed in lysis buffer and then analyzed by immunoblot. For immunoprecipitation of endogenous proteins, MKL1 cell lysates were precipitated with an anti-PP4R1 antibody or a pre-immune IgG control overnight at 4°C. Packed protein G beads (20 μl) were added to the lysates and antibody mix, and incubated for a further 1 hour at 4°C. Precipitates were washed extensively in lysis buffer and boiled in Laemmli loading buffer prior to SDS PAGE and western blot analysis.

### Luciferase reporter assays

MCC13 cells were transfected with 25 ng of reporter plasmid expressing firefly luciferase under the transcriptional control of the NF-κB elements from the concanavalin A promoter [42]. Where appropriate, cells were co-transfected with 1 μg of GFP or GFP-tAg expression plasmids. To normalize for transfection efficiency, 5 ng of pRLTK, which drives renilla luciferase expression from a constitutive TK promoter, was added to each transfection. At 24 h post transfection, cells were serum starved for 6 h and then treated with 10 ng/mL TNFα for a further 8 h. Samples were lysed in Passive Lysis Buffer (PLB) (Promega), and levels of luciferase detected using a dual luciferase reporter assay (Promega), as previously described [43]. For analysis of MCPyV early promoter activation, 200 ng of MCPyV promoter reporter plasmid was co-transfected with 1 μg of GFP or GFP-tAg expression plasmid and 5 ng pRLTK. Cells lysates were harvested after 24 hours and levels of luciferase determined as described above. Experiments were performed in triplicate and from at least three independent biological repeats.

### Determination of secreted IL-8 and CCL20 levels by ELISA

MCC13 cells expressing proteins of interest or treated with siRNA targeting PP4R1 were treated with 10 ng/ml TNF-α, and supernatants were collected 24 h post-treatment. Levels of secreted interleukin-8 (IL-8) and CCL20 were detected by enzyme-linked immunosorbent assay (ELISA) using the manufacturer's protocol (R&D Systems).

### Expression and purification of recombinant proteins

Recombinant GST, GST-tAg and GST-NEMO were expressed and purified using protocols previously described [23].

### *In vitro* pull-down assays

GST pull-down assays were performed as described previously [23, 44]. Briefly, GST fusion proteins were bound to glutathione-agarose beads at 4°C for 1 hour. Equal quantities of *in vitro* transcription/translation reactions (Promega TnT) expressing a protein of choice were incubated with beads in lysis buffer. After 3 hours of incubation the beads were washed extensively in lysis buffer, prior to analysis by SDS PAGE and Western blotting. GST alone served as a negative control.

## References

[1] Stakaitytė G, Wood JJ, Knight LM, Abdul-Sada H, Adzahar NS, Nwogu N, Macdonald A, Whitehouse A (2014). Merkel cell polyomavirus: molecular insights into the most recently discovered human tumour virus. Cancers (Basel).

[2] Schrama D, Ugurel S, Becker JC (2012). Merkel cell carcinoma: recent insights and new treatment options. Curr Opin Oncol.

[3] Feng H, Shuda M, Chang Y, Moore PS (2008). Clonal integration of a polyomavirus in human Merkel cell carcinoma. Science.

[4] Houben R, Angermeyer S, Haferkamp S, Aue A, Goebeler M, Schrama D, Hesbacher S (2015). Characterization of functional domains in the Merkel cell polyoma virus Large T antigen. Int J Cancer.

[5] Spurgeon ME, Cheng J, Bronson RT, Lambert PF, DeCaprio JA (2015). Tumorigenic activity of merkel cell polyomavirus T antigens expressed in the stratified epithelium of mice. Cancer Res.

[6] Shuda M, Kwun HJ, Feng H, Chang Y, Moore PS (2011). Human Merkel cell polyomavirus small T antigen is an oncoprotein targeting the 4E-BP1 translation regulator. J Clin Invest.

[7] Whitehouse A, Macdonald A (2015). Stathmin drives virus-induced metastasis. Oncotarget.

[8] Kwun HJ, Shuda M, Feng H, Camacho CJ, Moore PS, Chang Y (2013). Merkel cell polyomavirus small T antigen controls viral replication and oncoprotein expression by targeting the cellular ubiquitin ligase SCFFbw7. Cell Host Microbe.

[9] Knight LM, Stakaityte G, Wood JJ, Abdul-Sada H, Griffiths DA, Howell GJ, Wheat R, Blair GE, Steven NM, Macdonald A, Blackbourn DJ, Whitehouse A (2015). Merkel cell polyomavirus small T antigen mediates microtubule destabilization to promote cell motility and migration. J Virol.

[10] Griffiths DA, Abdul-Sada H, Knight LM, Jackson BR, Richards K, Prescott EL, Peach AH, Blair GE, Macdonald A, Whitehouse A (2013). Merkel cell polyomavirus small T antigen targets the NEMO adaptor protein to disrupt inflammatory signaling. J Virol.

[11] Kwun HJ, Shuda M, Camacho CJ, Gamper AM, Thant M, Chang Y, Moore PS (2015). Restricted protein phosphatase 2A targeting by Merkel cell polyomavirus small T antigen. J Virol.

[12] Richards KH, Macdonald A (2011). Putting the brakes on the anti-viral response: negative regulators of type I interferon (IFN) production. Microbes Infect.

[13] Perkins ND (2007). Integrating cell-signalling pathways with NF-kappaB and IKK function. Nat Rev Mol Cell Biol.

[14] Adhikari A, Xu M, Chen ZJ (2007). Ubiquitin-mediated activation of TAK1 and IKK. Oncogene.

[15] Clark K, Nanda S, Cohen P (2013). Molecular control of the NEMO family of ubiquitin-binding proteins. Nat Rev Mol Cell Biol.

[16] Emmerich CH, Ordureau A, Strickson S, Arthur JS, Pedrioli PG, Komander D, Cohen P (2013). Proceedings of the National Academy of Sciences..

[17] DeCaprio JA, Garcea RL (2013). A cornucopia of human polyomaviruses. Nat Rev Microbiol.

[18] Ku CL, Picard C, Erdös M, Jeurissen A, Bustamante J, Puel A, von Bernuth H, Filipe-Santos O, Chang HH, Lawrence T, Raes M, Maródi L, Bossuyt X (2007). IRAK4 and NEMO mutations in otherwise healthy children with recurrent invasive pneumococcal disease. J Med Genet.

[19] Filipe-Santos O, Bustamante J, Haverkamp MH, Vinolo E, Ku C-L, Puel A, Frucht DM, Christel K, von Bernuth H, Jouanguy E, Feinberg J, Durandy A, Senechal B (2006). X-linked susceptibility to mycobacteria is caused by mutations in NEMO impairing CD40-dependent IL-12 production. J. Exp Med..

[20] Rahighi S, Ikeda F, Kawasaki M, Akutsu M, Suzuki N, Kato R, Kensche T, Uejima T, Bloor S, Komander D, Randow F, Wakatsuki S, Dikic I (2009). Specific recognition of linear ubiquitin chains by NEMO is important for NF-kappaB activation. Cell.

[21] Vinolo E, Sebban H, Chaffotte A, Israël A, Courtois G, Véron M, Agou F (2006). A point mutation in NEMO associated with anhidrotic ectodermal dysplasia with immunodeficiency pathology results in destabilization of the oligomer and reduces lipopolysaccharide- and tumor necrosis factor-mediated NF-kappa B activation. J Biol Chem.

[22] Temmerman ST, Ma CA, Borges L, Kubin M, Liu S, Derry JM, Jain A (2006). Impaired dendritic-cell function in ectodermal dysplasia with immune deficiency is linked to defective NEMO ubiquitination. Blood.

[23] Macdonald A, Crowder K, Street A, McCormick C, Harris M (2004). The hepatitis C virus NS5A protein binds to members of the Src family of tyrosine kinases and regulates kinase activity. J Gen Virol.

[24] Kloeker S, Wadzinski BE (1999). Purification and identification of a novel subunit of protein serine/threonine phosphatase 4. J Biol Chem.

[25] Hwang JH, Pores Fernando AT, Faure N, Andrabi S, Adelmant G, Hahn WC, Marto JA, Schaffhausen BS, Roberts TM (2014). Polyomavirus small T antigen interacts with yes-associated protein to regulate cell survival and differentiation. J Virol.

[26] Kawai T, Akira S (2007). SnapShot: Pattern-Recognition Receptors. Cell.

[27] Rahman MM, McFadden G (2011). Modulation of NF-kappa B signalling by microbial pathogens. Nature Publishing Group.

[28] DiPerna G, Stack J, Bowie AG, Boyd A, Kotwal G, Zhang Z, Arvikar S, Latz E, Fitzgerald KA, Marshall WL (2004). Poxvirus protein N1L targets the I-kappaB kinase complex, inhibits signaling to NF-kappaB by the tumor necrosis factor superfamily of receptors, and inhibits NF-kappaB and IRF3 signaling by toll-like receptors. J Biol Chem.

[29] Brady G, Bowie AG (2014). Innate immune activation of NFκB and its antagonism by poxviruses. Cytokine Growth Factor Rev.

[30] Mohamed MR, McFadden G (2009). NFkB inhibitors: strategies from poxviruses. Cell Cycle.

[31] Kensche T, Tokunaga F, Ikeda F, Goto E, Iwai K, Dikic I (2012). Analysis of nuclear factor-κB (NF-κB) essential modulator (NEMO) binding to linear and lysine-linked ubiquitin chains and its role in the activation of NF-κB. J Biol Chem.

[32] Laplantine E, Fontan E, Chiaravalli J, Lopez T, Lakisic G, Véron M, Agou F, Israël A (2009). NEMO specifically recognizes K63-linked poly-ubiquitin chains through a new bipartite ubiquitin-binding domain. EMBO J.

[33] Hubeau M, Ngadjeua F, Puel A, Israel L, Feinberg J, Chrabieh M, Belani K, Bodemer C, Fabre I, Plebani A, Boisson-Dupuis S, Picard C, Fischer A (2011). New mechanism of X-linked anhidrotic ectodermal dysplasia with immunodeficiency: impairment of ubiquitin binding despite normal folding of NEMO protein. Blood.

[34] Nanda SK, Venigalla RK, Ordureau A, Patterson-Kane JC, Powell DW, Toth R, Arthur JS, Cohen P (2011). Polyubiquitin binding to ABIN1 is required to prevent autoimmunity. J Exp Med.

[35] Lo YC, Lin SC, Rospigliosi CC, Conze DB, Wu CJ, Ashwell JD, Eliezer D, Wu H (2009). Structural basis for recognition of diubiquitins by NEMO. Mol Cell.

[36] Hadweh P, Habelhah H, Kieff E, Mosialos G, Hatzivassiliou E (2014). The PP4R1 subunit of protein phosphatase PP4 targets TRAF2 and TRAF6 to mediate inhibition of NF-κB activation. Cell Signal.

[37] Brechmann M, Mock T, Nickles D, Kiessling M, Weit N, Breuer R, Müller W, Wabnitz G, Frey F, Nicolay JP, Booken N, Samstag Y, Klemke CD (2012). A PP4 holoenzyme balances physiological and oncogenic nuclear factor-kappa B signaling in T lymphocytes. Immunity.

[38] Chen L, Dong W, Zou T, Ouyang L, He G, Liu Y, Qi Y (2008). Protein phosphatase 4 negatively regulates LPS cascade by inhibiting ubiquitination of TRAF6. FEBS Lett.

[39] Langeberg LK, Scott JD (2015). Signalling scaffolds and local organization of cellular behaviour. Nat Rev Mol Cell Biol.

[40] Richards KH, Doble R, Wasson CW, Haider M, Blair GE, Wittmann M, Macdonald A (2014). Human papillomavirus E7 oncoprotein increases production of the anti-inflammatory interleukin-18 binding protein in keratinocytes. J Virol.

[41] Müller M, Wasson CW, Bhatia R, Boxall S, Millan D, Goh GY, Haas J, Stonehouse NJ, Macdonald A (2015). YIP1 family member 4 (YIPF4) is a novel cellular binding partner of the papillomavirus E5 proteins. Sci Rep.

[42] Gamlen T, Richards KH, Mankouri J, Hudson L, McCauley J, Harris M, Macdonald A (2010). Expression of the NS3 protease of cytopathogenic bovine viral diarrhea virus results in the induction of apoptosis but does not block activation of the beta interferon promoter. J Gen Virol.

[43] Mankouri J, Fragkoudis R, Richards KH, Wetherill LF, Harris M, Kohl A, Elliott RM, Macdonald A (2010). Optineurin negatively regulates the induction of IFNbeta in response to RNA virus infection. PLoS Pathog.

[44] Macdonald A, Mazaleyrat S, McCormick C, Street A, Burgoyne NJ, Jackson RM, Cazeaux V, Shelton H, Saksela K, Harris M (2005). Further studies on hepatitis C virus NS5A-SH3 domain interactions: identification of residues critical for binding and implications for viral RNA replication and modulation of cell signalling. J Gen Virol.

